# Frailty Was Associated With Atmospheric NO_2_ Levels: A Geospatial Approach

**DOI:** 10.1093/gerona/glae168

**Published:** 2024-07-03

**Authors:** Emmanuel Gonzalez-Bautista, Susana Román-Pérez, Betty Soledad Manrique-Espinoza, Aaron Salinas-Rodríguez, René Santos-Luna

**Affiliations:** Center for Evaluation and Survey Research, National Institute of Public Health (INSP), Cuernavaca, Morelos, Mexico; Maintain Aging Research Team, CERPOP, Inserm, Université Paul Sabatier, Université de Toulouse, Toulouse, France; Center for Evaluation and Survey Research, National Institute of Public Health (INSP), Cuernavaca, Morelos, Mexico; Center for Evaluation and Survey Research, National Institute of Public Health (INSP), Cuernavaca, Morelos, Mexico; Center for Evaluation and Survey Research, National Institute of Public Health (INSP), Cuernavaca, Morelos, Mexico; Center for Evaluation and Survey Research, National Institute of Public Health (INSP), Cuernavaca, Morelos, Mexico

**Keywords:** Air quality, Frailty, GIS, Older adults, Satellite data

## Abstract

**Background:**

Recent evidence has linked air pollution with frailty, yet little is known about the role of NO_2_ in this association. Our aim was to assess the association between frailty and NO_2_ air concentrations in Mexican older adults.

**Methods:**

We used georeferenced data from the population-based Nutrition and Health Survey in Mexico (NHNS) 2021, representative of national and subnational regions, to measure a frailty index based on 31 health deficits in adults aged 50 and older. Air pollution due to NO_2_ concentrations was estimated from satellite images validated with data from surface-level stations. Maps were produced using Jensen’s Natural break method. The association of frailty and NO_2_ concentrations was measured using the frailty index (multivariate fractional response logit regression) and a frailty binary variable (frailty index [FI] ≥0.36, multivariate logit regression).

**Results:**

There was a positive and significant association of the frailty index with the NO_2_ concentrations, adjusting for age, sex, urban and rural area, years of education, socioeconomic status, living arrangement, particulate matter smaller than 2.5 microns, and indoor pollution. For each standard deviation increase in NO_2_ concentrations measured 10 years before the survey, the odds of being frail were 15% higher, and the frailty index was 14.5% higher. The fraction of frailty attributable to NO_2_ exposure ranged from 1.8% to 23.5% according to different scenarios.

**Conclusions:**

Frailty was positively associated with exposure to NO_2_ concentrations. Mapping frailty and its associated factors like NO_2_ air concentrations can contribute to the design of targeted pro-healthy aging policies.

Air pollution has been recognized as a pressing threat to functioning in older adults (1). The proportion of the aging population living in urban areas is growing (2). Environmental factors related to frailty are thus becoming increasingly relevant to stakeholders in pursuing healthy aging (3). Frailty, a geriatric syndrome characterized by high vulnerability to stressors and diminished physiological reserve, is a suitable outcome to explore multisystemic perturbations potentially caused by cumulative exposure to harmful stimuli of diverse nature (eg, environmental factors) (4,5).

Among the myriad of frailty assessment methods, the frailty index complies with the deficit accumulation approach and evaluates impairments across biological systems (5). Individual-level biomedical frailty correlates have been widely described (6). Nevertheless, few studies have explored the correlates of frailty from a geospatial approach (3,7,8). For example, satellite data on atmospheric pollutants like ozone and nitrogen dioxide has recently become available (9–11).

Similarly, the association between frailty and air pollution has been recently reported (3,12). A few studies have found an association between frailty and fine particulate matter (PM2.5), but evidence of the link with other pollutants like NO_2_ is scarce (13).

Natural NO_2_ emissions have a very low background concentration. Human activity-related emissions are the most significant factor in human exposure. The primary cause of NO_2_ emissions are anthropogenic combustion processes (for heat or electricity generation or in combustion engines). For instance, the main sources of nitrogen oxides are road-transport vehicles (14).

NO_2_ concentrations are associated with increased disease and mortality risks even after adjusting for socioeconomic status and other potential confounders (15–17). Short-term exposures to peak air pollution levels are associated with higher emergency visits due to system-specific outcomes such as respiratory diseases (18,19).

On the other hand, long-term exposure to NO_2_ is associated with health impacts, notably in the respiratory system (higher risk for incident adult asthma (20) and chronic obstructive pulmonary disease) (21). Yet, extra-pulmonary effects have also been reported regarding cardiovascular events (higher risk to transition from prehypertension to hypertension, and then to cardiovascular disease and death) (22), neurological features (lower cortical thickness in Alzheimer’s disease-related brain regions) (23), and incident diabetes (24). In older adults, these pathologies could explain the link of NO_2_ with frailty. However, to the best of our knowledge, no direct association with muscle-disturbing pathophysiology has been investigated yet (25). Long-term NO_2_ exposure has been linked to cardiovascular and respiratory disease, cancer, ischemic heart disease, and cerebrovascular disease mortality in older people in the United States (26). Yet, little is known about the association of long-term exposure to NO_2_ and an integrative marker of (un)healthy aging such as frailty.

We hypothesize that long-term exposure to high levels of other air pollutants, such as NO_2,_ will be associated with a higher frailty index in older people. This study aimed to assess the association between frailty and NO_2_ air concentrations in older adults.

## Method

The NHNS was approved by the institutional review board of the National Institute of Public Health in Mexico (CI-450-2021). All participants signed informed consent before responding.

### Study Population

We used data from the National Health and Nutrition Survey (NHNS) 2021 in Mexico. NHNS is a survey with national and subnational representativeness of community-dwelling adults by multistage cluster sampling. The primary sampling units were the standard geostatistical units called AGEB, which allowed for the geospatial analysis of data. The AGEB is an urban or rural territorial extension that standardizes the statistical information from censuses and surveys carried out by the National Institute of Geography and Statistics in Mexico. We included people aged 50 and older as respondents of a household survey. Methodological details of the NHNS have been published elsewhere (27).

### Frailty Index

Following standard procedures (28) and previous work (29), we selected 31 age-related items from the NHNS questionnaire. We scored 1 for each accumulated deficit and divided the sum by 31 (or the number of nonmissing items). The maximum theoretical frailty was frailty index (FI) = 1.0. We verified that the FI was log-related with chronological age. To check for selection bias, we calculated a version of the frailty index, omitting the items related to access to healthcare (eg, “Has a doctor told you that you have diabetes [or high blood sugar]?”). There were no significant differences among these 2 versions, which suggests that the 31-item frailty index is not biased by healthcare access. We included all participants with at least 29 out of 31 items of the frailty index (95% of nonmissing FI items). Details on the items included in the frailty index are available in Supplementary Material S1.

Binary frailty was defined as FI ≥0.36 (30).

### Nitrogen Dioxide

We used NASA’s Health and Air Quality Applied Sciences Team (HAQAST) data set of yearly mean NO_2_ concentrations at surface-level V1 (SFC_NITROGEN_DIOXIDE_CONC) (31), from 2011 to 2020 measured in ppb. The yearly average concentrations and the period averages can be found in Supplementary Table S2. Satellite data were produced based on 5 220 NO_2_ monitors in 58 countries and land use variables, with a resolution of 0.00083° (~1 km^2^) via land use regression methods to determine spatial variation at local scales and has undergone validation vis-a-vis ground stations to predict monthly and annual averages of NO_2_ (31–36).

We used the AGEB level to attribute the NO_2_ concentrations to the NHNS participants living in that AGEB. We calculated the mean NO_2_ concentration for 10 periods (from 10 to 1 year before the survey) as the main exposure variable. To account for variations in the AGEBs’ surface according to population density, we estimated the population-weighted average of NO_2_ concentrations.

Using Jensen’s natural break method, ArcGIS 10.6 was used for data transfer, georeferencing, and map production. QGIS 3.4 was used to import the products (SFC_NITROGEN_DIOXIDE_CONC) and to transform netCDF to TIFF format.

### Covariates

The concentration of the particles with a diameter <2.5 microns (PM2.5) was obtained from NASA’s HAQAST product, TOTSMASS25. We proceeded alike with the NO_2_, calculating the mean NO_2_ concentration for 10 periods (from 10 to 1 year before the survey) as a covariate.

Besides, we included age, sex, urban and rural area, education as the number of years of formal education, living arrangement (living alone = 1, otherwise = 0), an index of household assets as a proxy of household wealth, and a proxy for indoor pollution based on the use of fossil fuels without ventilation or an extraction facility.

### Statistical Analyses

We visually inspected the data distribution using histograms and scatterplots. We used means and proportions to describe the study population (Table 1).

**Table 1.** Description of the Population

Frail (Frailty Index ≥ 0.36)			
*n* (%) or Mean (*SD*)	No	Yes	Total
*N*	1 568 (78.1%)	439 (21.9%)	2 007 (100.0%)
Age, y	72.7 (6.6)	74.1 (7.0)	73.0 (6.7)^*^
Female	869 (55.4%)	312 (71.1%)	1 181 (58.8%)^*^
Urban and metropolitan area	1 128 (75.7%)	312 (75.0%)	1 440 (75.5%)
Wealth index	1.4 (1.1)	1.3 (1.0)	1.4 (1.1)^*^
Years of education	7.7 (5.6)	6.6 (4.9)	7.5 (5.5)^*^
Living alone	396 (25.3%)	121 (27.6%)	517 (25.8%)
Frailty index	0.207 (0.089)	0.459 (0.071)	0.262 (0.134)^*^
NO_2_ average for *x* years before survey, ppb			
1 y	9.7 (4.5)	10.0 (4.5)	9.8 (4.525)
5 y	10.4 (5.0)	10.7 (5.0)	10.5 (5.009)
10 y	10.9 (5.6)	11.3 (5.7)	11.0 (5.649)

*Notes*: ppb = parts per billion.

^*^Between group differences at the .05 level of significance.

For each of the 10 periods considered (from 10 to 1 year before the survey), we modeled the association between NO_2_ and frailty using a multivariate logit regression with binary frailty defined as FI ≥0.36. The association with the continuous FI was also investigated as sensitivity analyses with multivariate fractional response logit regression.

To render the results more interpretable, we estimated the marginal probability of being frail for each 1 *SD* change in NO_2_ concentration for the period of 10 years before the NHNS 2021 survey and similarly for the change in the FI.

All models were adjusted for age, sex, urban and rural area, education, household asset index, living arrangement, indoor pollution, and the corresponding PM2.5 average concentrations for each period. We verified the VIF values for our models to discard multicollinearity.

Additionally, we estimated the population-attributable fraction based on 2 pairs of hypothetical scenarios: (A) achieving the complete application of the current norms, that is, lowering NO_2_ exposure to lower than 21 ppb, and (B) lowering NO_2_ exposure from higher quartiles to the lowest observed quartile (Q2 to Q1, Q3 to Q1, and Q4 to Q1). We used the punaf package. Statistical analyses were performed in Stata 18, StataCorp. LLC, 2023, College Station, TX, USA.

## Results

Our population included 2 007 participants aged 50+ with data for the frailty index (95% of nonmissing FI items), with a mean age of 73.0 (*SD* = 6.7); 58.8% were female, and 21.9% were frail. The mean frailty index for the population aged 50 and older was 0.26 (*SD* = 0.13). The 2020 population-weighted average concentration of NO_2_ in Mexico was 9.8 ppb, and for the study period (2011–2020), the population-weighted average was 11.5 ppb in urban and 4.1 in rural areas.

The analytic sample included 1 907 adults aged 50+ with complete data for the covariates.

### Main Results

The odds of being frail increased significantly per each ppb increase in NO_2_ concentrations, adjusting for age, sex, rural and urban localization, education, wealth index, living alone, PM2.5 concentrations, and indoor pollution (Table 2). This association was significant for the NO_2_ concentrations dated 2 or more years before the NHNS in 2021 (Table 2). In other words, for each standard deviation increase in NO_2_ concentrations (average NO_2_ concentrations of 10 years before the survey), the odds of being frail were 15% higher. This translates to the adjusted marginal probabilities of being frail ranging from 0.21 for people exposed to mean NO_2_ levels to 0.26 for people exposed to NO_2_ levels 3 *SD* above the mean (Supplementary Figure S1). The VIF values ranged from 1.02 to 1.30, with a mean VIF of 1.11, with negligible impact of collinearity in our models.

**Table 2.** Association Between NO_2_ and Frailty

*n* = 1 907 *Main Analyses*				
Binary Frailty (FI >0.36)	OR	*p* Value	95% CI lb	95% CI ub
Average of *x* years previous to survey				
Ten	1.025	.022	1.004	1.047
Nine	1.025	.026	1.003	1.048
Eight	1.025	.03	1.002	1.048
Seven	1.025	.035	1.002	1.049
Six	1.025	.039	1.001	1.050
Five	1.025	.04	1.001	1.050
Four	1.026	.039	1.001	1.051
Three	1.027	.041	1.001	1.053
Two	1.027	.048	1.000	1.055
One	1.025	.065	0.998	1.053
*Sensitivity Analyses*				
Frailty Index	Coef.	*p* Value	95% CI lb	95% CI ub
Average of *x* years previous to survey				
Ten	0.007	.023	0.001	0.013
Nine	0.007	.029	0.001	0.013
Eight	0.007	.036	0.000	0.013
Seven	0.006	.045	0.000	0.013
Six	0.006	.053	0.000	0.013
Five	0.006	.057	0.000	0.013
Four	0.006	.058	0.000	0.013
Three	0.006	.066	0.000	0.013
Two	0.006	.083	−0.001	0.014
One	0.006	.127	−0.002	0.013

*Notes*: Coef = coefficient from linear regression interpreted as the change in the frailty index (0–1) per each increase in 1 ppb of NO_2_ average exposure in the period.

All models adjusted for age, sex, rural and urban localization, education, wealth index, living alone, PM2.5 concentrations, and indoor pollution.

### Sensitivity Analyses

The estimated change in the FI ranged between 0.006 and 0.007 per each increase in 1 ppb of NO_2_. In other words, for each standard deviation increase in NO_2_ concentrations, the frailty index was 14.5% higher, and the odds of being frail were 15% higher (average NO_2_ concentrations of 10 years before the survey). The association was significant for the NO_2_ concentration averages dated 2 or more years before the NHNS 2021 for binary frailty and 7 or more years before the NHNS 2021 for the FI. This translates to the adjusted marginal frailty index ranging from 0.26 for people exposed to mean NO_2_ levels to 0.28 for people exposed to NO_2_ levels 3 *SD* above the mean (Supplementary Figure S1).

**Figure 1.**
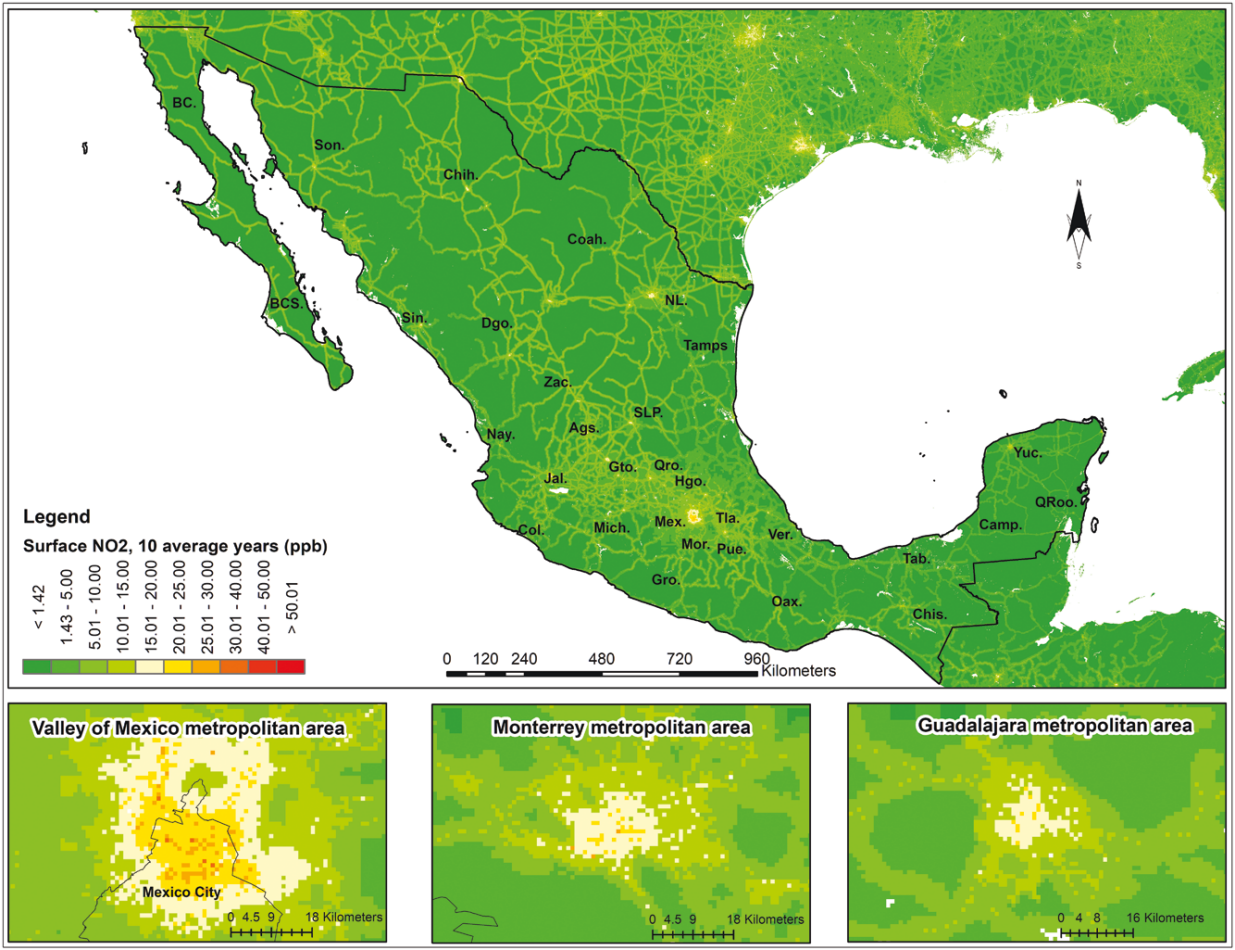
Geospatial distribution of the average nitrogen dioxide concentrations from 2011 to 2020 in Mexico.

**Figure 2.**
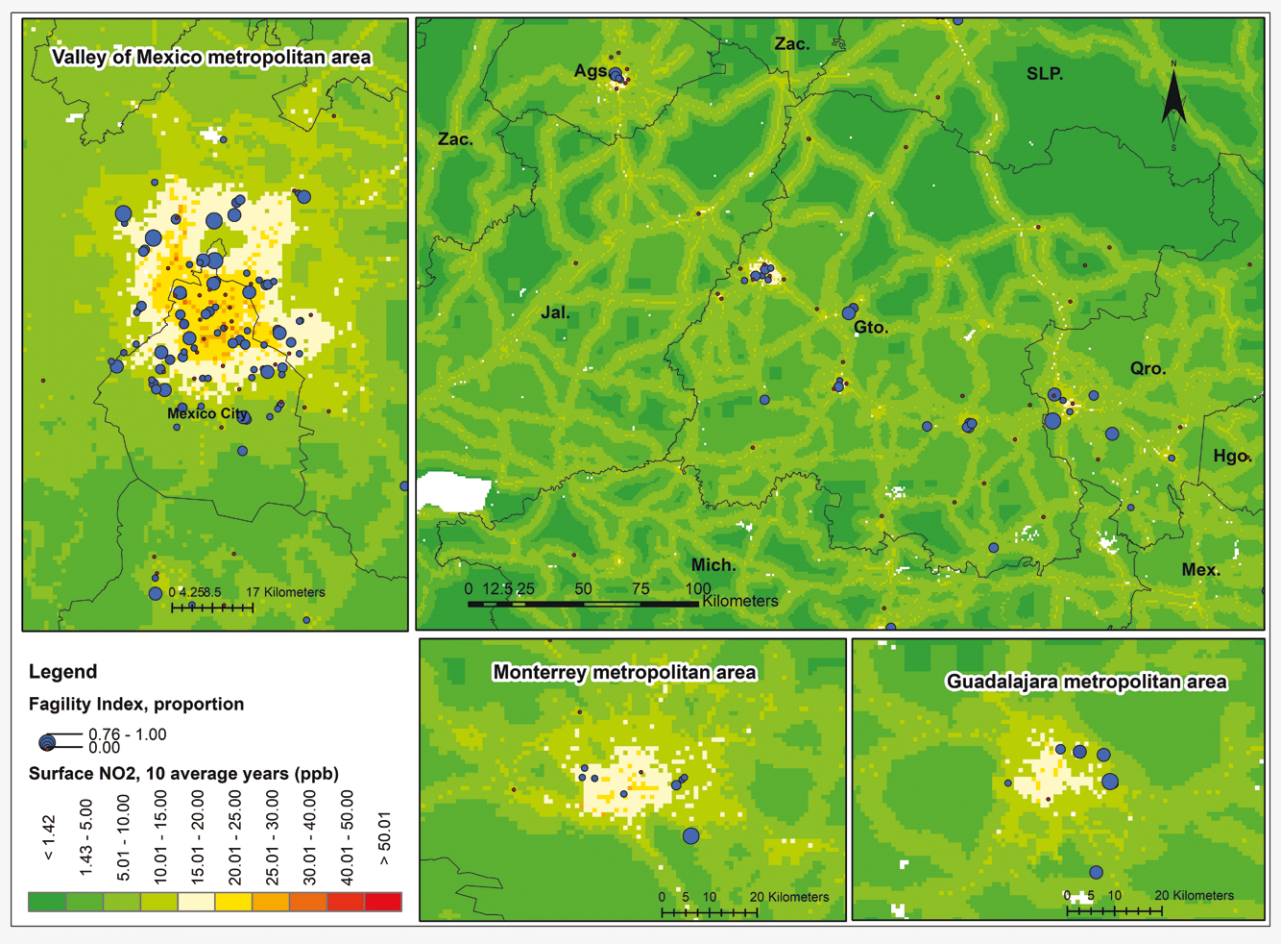
Overlapping of NO_2_ average concentrations and frailty in rural and urban areas in Mexico.

The highest NO_2_ concentrations were in the major urban areas of Mexico City, Guadalajara, and Monterrey. The maps showed high NO_2_ concentrations along the highways connecting urban areas (Figure 1). Rural or unpopulated areas showed shallow NO_2_ levels. The frailty index tended to show high values in areas with high NO_2_ concentrations (Figure 2). However, the maps also revealed participants with high FI values living in the peripheral areas of major urban concentrations.

### Population-Attributable Fraction

**Figure 3.**
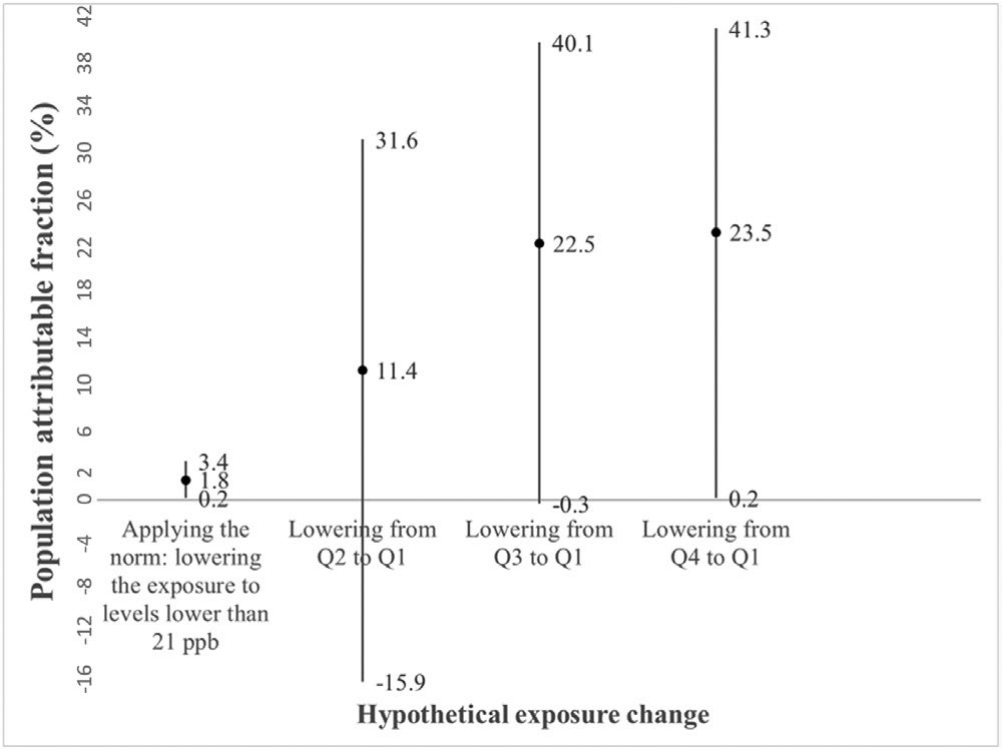
Population-attributable fractions according to hypothetical NO_2_ exposure changes.

The population-attributable fraction under scenario (A) achieving the complete application of the current norms, that is lowering NO_2_ exposure to lower than 21 ppb was PAF = 1.8% (95% CI: 0.2; 3.4), and (B) lowering NO_2_ exposure from higher quartiles to the lowest observed quartile: PAF_Q2 to Q1_ = 11.4% (95% CI: −15.9; 31.6), PAF_Q3 to Q1_ = 22.5% (95% CI: −0.3; 40.1), and PAF_Q4 to Q1_ = 23.5% (95% CI: 0.2; 41.3) (Figure 3).

## Discussion

Our study found a positive and significant association of past exposure to NO_2_ concentrations measured via satellite imaging with earth-level validation and the odds of being frail (FI ≥0.36) adjusting for age, sex, education, urban and rural area, wealth index, living arrangement, indoor pollution, and PM2.5 concentrations. Sensitivity analyses revealed a consistent association with the continuous frailty index (measured with a frailty index of 31 health traits).

Previous work had shown an association of long-term exposure (at least 1 year before the frailty measurement) to air pollution with frailty in older adults in China (37). Moreover, Veronese and co-authors (13) showed an association between various nitrogen oxides (along with particulate matter) and frailty measured using the 5 Fried’s frailty criteria (38). Our results align with such findings with a robust yet modest association.

We think such a link can be due to a consistent yet nonlinear association of frailty with air pollution and is rather indirect in the causal network, with a more direct association for people with respiratory diseases. An alternative mechanism could be systemic inflammation related to chronic air pollution exposure, which affects cardiovascular and cognitive function and may lead to frailty (39). For instance, a recent study has found a link between NO_2_ and other air pollutants with all-cause dementia, and also separately for vascular dementia and Alzheimer’s disease (40).

The observed average NO_2_ concentration in Mexico during our study period (2011–2020) was 9.8 ppb, exceeding the international Air Quality Guideline (AQG) recommendation of 5.3 ppb (41). The primary sources of NO_2_ in Mexico are fossil fuel combustion from transportation and industrial activities (35), with additional impact from unclean cooking fuel use (42). Although current local regulations set a yearly average limit of 21 ppb (43), our research suggests that a lower threshold of 5 ppb or less may be more appropriate to mitigate the potential health risks, especially considering recent meta-analyses associating NO_2_ with increased mortality, including chronic obstructive pulmonary disease (44).

Our work has strengths, such as using recently released satellite-based NO_2_ concentration data, whose estimation has been validated by earth-level sensing stations. In addition, to the best of our knowledge, we present the first study with georeferenced data that allows for mapping frailty cases and their overlap with NO_2_ atmospheric concentrations using data at the country level in Latin America. Our study has limitations; for instance, we could not retrieve past frailty measures from previous NHNSs, given the absence of items needed for the frailty index, and the differences in sampling units impeding the georeferentiation. Therefore, we had to assume that the participants whose frailty was measured in 2021 had been exposed to their geographically attributed NO_2_ concentrations throughout the period before being surveyed. Although we controlled for several strong frailty determinants and other sources of air pollution (indoor and PM 2.5), our results are subject to residual confounding (eg, rural–urban migration during the life course). Also, the NHNS 2021 did not include a measurement of muscle strength or muscle power, impeding us to further characterize the level of muscle-related function in our study.

### Perspectives and Mitigation Strategies

Our research highlights a significant association between frailty in older adults and NO_2_ concentrations in a developing country like Mexico. As urbanization and population aging continue to shape public health challenges, a geospatial approach provides valuable insights into the geographical distribution of frailty in developing countries. More research is needed to elucidate the underlying mechanisms of this association. Next rounds of the NHNS should include measurements of muscle strength or muscle power (ie, the chair stand test or grip strength) to allow for a deeper understanding of the association of NO_2_ and frailty in older adults.

Mitigation strategies should be implemented at different scales. From a population level, countries should consider the WHO’s recent recommendations. The WHO Guidelines endorse that the current maximal yearly mean exposure of 40 µg/m^3^ is progressively lowered to 10 µg/m^3^, with interim targets of 30 and then 20 µg/m^3^ (45). On the other hand, home-based mitigation steps to reduce indoor exposure include not idling the car inside the garage, properly adjusting gas appliances, installing and using an exhaust fan vented outdoors over gas stoves, and having a trained professional inspect, clean, and tune up central heating systems annually. Finally, further research is needed to reveal individual-level interventions to mitigate the effects of chronic NO_2_ exposure (eg, potentially by increasing the antioxidants intake).

## Conclusion

Chronic exposure to high NO_2_ levels was associated with higher frailty prevalence. Innovative approaches to healthy aging can benefit from the use of satellite-derived data.

## Supplementary Material

glae168_suppl_Supplementary_Materials
