# Supplementary material for: Frailty Was Associated With Atmospheric NO2 Levels: A Geospatial Approach
Source: J Gerontol A Biol Sci Med Sci. 2024 Jul 3;79(9):glae168. doi: 10.1093/gerona/glae168 (PMC11333824; doi:10.1093/gerona/glae168)
Supplement: glae168_suppl_Supplementary_Materials [file glae168_suppl_supplementary_materials.pdf]

Supplementary Table S1. Items in the frailty index

1.	Has a doctor told you that you have diabetes (or high blood sugar)?
2.	Has a doctor or other health care professional ever told you that you suffer, or have you suffered from depression?
3.	Has a doctor told you that you have high blood pressure?
4.	Has the doctor told you that you have (or had) a myocardial infraction or heart attack?
5.	Has your doctor told you if you have had a stroke?
6.	Has a doctor told you that you have high cholesterol?
7.	Has a doctor told you that you have high triglycerides?
8.	Has a doctor ever told you that you have a urinary tract infection on more than one occasion?
9.	Has a doctor ever told you that you have kidney stones?
10.	Has a doctor ever told you that you have kidney failure or chronic kidney disease?
11.	Have you suffered any damage to your health due to an accident in the last 12 months?
12.	Have you ever thought about suicide?
13.	Do you wear glasses or contact lenses?
14.	Have you lost weight during the last 12 months?
15.	Did you feel like you couldn't shake the sadness?
16.	Was it difficult for you to concentrate on what you were doing?
17.	Have you felt down or depressed?
18.	Did it seem like everything you did was an effort?
19.	Have you had troubles to sleep well?
20.	Have you enjoyed the life?
21.	Have you felt sad?
22.	Do you have difficulty hearing?
23.	Do you have difficulty walking or climbing steps?
24.	Do you have difficulty remembering or any memory problem?
25.	Do you have difficulty with self-care, such as washing your entire body or dressing?
26.	Using your native language, do you have difficulty communicating, for example understanding others or making yourself understood?
27.	Do you currently smoke tobacco?
28.	In the past 12 months, how often did you have at least one glass of wine, beer, whiskey, or any other beverage containing alcohol? (Daily, once every week or once every month)
29.	How often do you feel isolated from others? (Sometimes or frequently)
30.	How many times in the past week did you spend time with someone who doesn't live with you, that is, you went to visit someone or they came to visit you, or you went out together? (Never or almost never)
31.	How often do you feel left out? (Sometimes or frequently)

Supplementary Table S2. Yearly average levels and cumulative NO_2 levels

	NO_2 concentrations (ppb)	
Year	Yearly average	Cummulative average
2020	9.78	9.78
2019	10.11	9.95
2018	10.73	10.21
2017	10.95	10.39
2016	10.92	10.50
2015	10.96	10.58
2014	11.12	10.65
2013	11.51	10.76
2012	11.82	10.88
2011	12.32	11.02

Supplementary Fig S1. Marginal probability of being frail according to standardized values of NO2 concentrations 10 years before the NHNS.

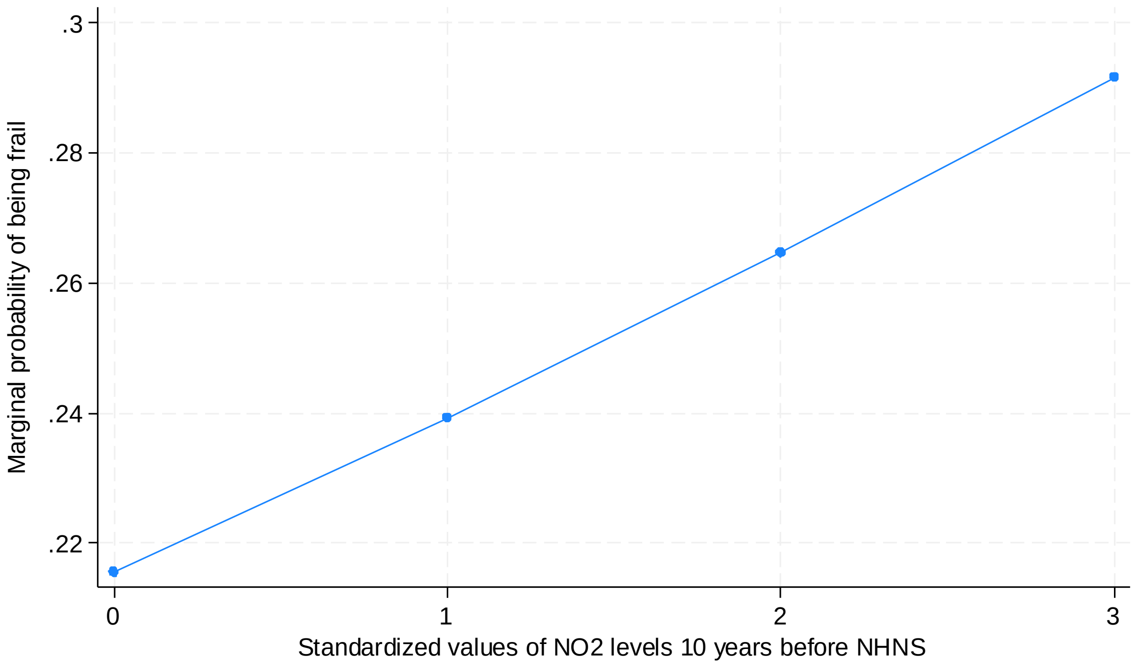

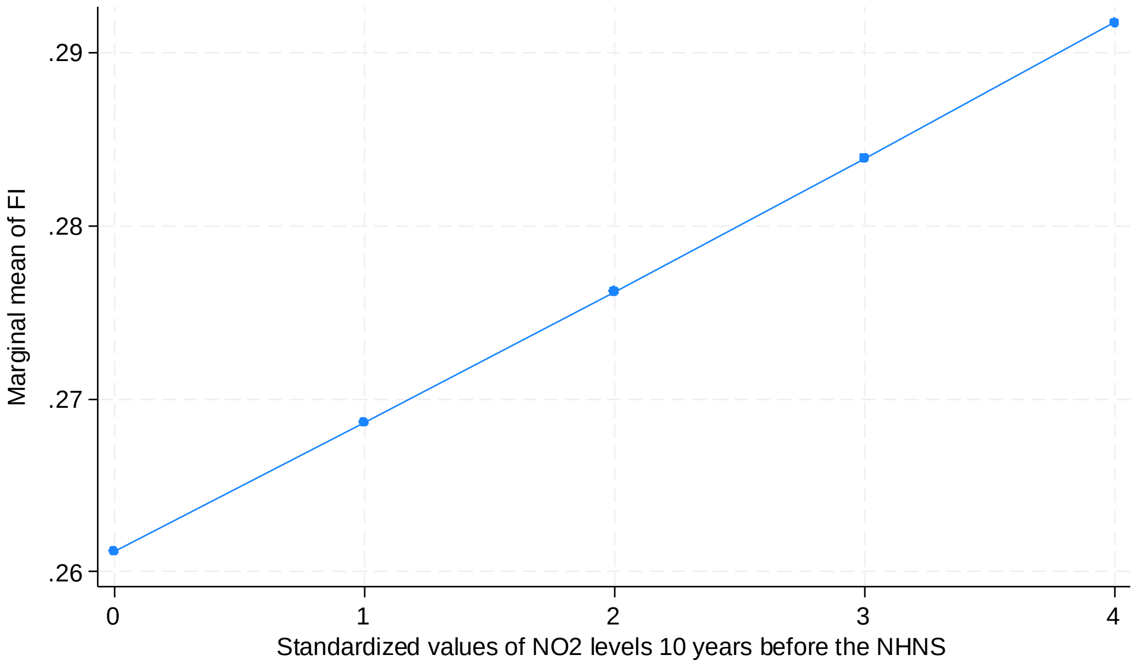

Marginal mean of FI
.26
.27
.28
.29
0
1
2
3
4
Standardized values of NO2 levels 10 years before the NHNS
